# Predictive Maintenance of Pins in the ECD Equipment for Cu Deposition in the Semiconductor Industry

**DOI:** 10.3390/s23146249

**Published:** 2023-07-08

**Authors:** Umberto Amato, Anestis Antoniadis, Italia De Feis, Domenico Fazio, Caterina Genua, Irène Gijbels, Donatella Granata, Antonino La Magna, Daniele Pagano, Gabriele Tochino, Patrizia Vasquez

**Affiliations:** 1 Institute of Applied Sciences and Intelligent Systems, National Research Council of Italy, 80131 Naples, Italy; antoniadis.anestis@na.isasi.cnr.it; 2 Institute of Applied Calculus, National Research Council of Italy, 80131 Naples, Italy; i.defeis@iac.cnr.it; 3 STMicroelectronics, Catania Wafer Fab Operations, 95121 Catania, Italy; caterina.genua@st.com (C.G.); gabriele.tochino@st.com (G.T.); domenico.fazio@st.com (D.F.); daniele.pagano@st.com (D.P.); patrizia.vasquez@st.com (P.V.); 4 Department of Mathematics, Katholieke Universiteit Leuven, 3001 Leuven, Belgium; irene.gijbels@kuleuven.be; 5 Institute of Applied Calculus, National Research Council of Italy, 00185 Rome, Italy; donatella.granata@cnr.it; 6 Institute of Microelectronics and Microsystems, National Research Council of Italy, 95121 Catania, Italy; antonino.lamagna@imm.cnr.it

**Keywords:** semiconductors, Predictive Maintenance, image processing, optical sensor

## Abstract

Nowadays, Predictive Maintenance is a mandatory tool to reduce the cost of production in the semiconductor industry. This paper considers as a case study a critical part of the electrochemical deposition system, namely, the four Pins that hold a wafer inside a chamber. The aim of the study is to replace the schedule of replacement of Pins presently based on fixed timing (Preventive Maintenance) with a Hardware/Software system that monitors the conditions of the Pins and signals possible conditions of failure (Predictive Maintenance). The system is composed of optical sensors endowed with an image processing methodology. The prototype built for this study includes one optical camera that simultaneously takes images of the four Pins on a roughly daily basis. Image processing includes a pre-processing phase where images taken by the camera at different times are coregistered and equalized to reduce variations in time due to movements of the system and to different lighting conditions. Then, some indicators are introduced based on statistical arguments that detect outlier conditions of each Pin. Such indicators are pixel-wise to identify small artifacts. Finally, criteria are indicated to distinguish artifacts due to normal operations in the chamber from issues prone to a failure of the Pin. An application (PINapp) with a user friendly interface has been developed that guides industry experts in monitoring the system and alerting in case of potential issues. The system has been validated on a plant at STMicroelctronics in Catania (Italy). The study allowed for understanding the mechanism that gives rise to the rupture of the Pins and to increase the time of replacement of the Pins by a factor at least 2, thus reducing downtime.

## 1. Introduction

Industry 4.0, or the fourth industrial revolution, promises a new paradigm for manufacturing based on the heavy use of cyber–physical systems, big data analytics, and Artificial Intelligence (AI) [1]. The semiconductor industry, which is highly innovative, is prone to benefit most from it in terms of efficiency, quality, and competitiveness. It develops and produces chips for computers, smartphones, and in general all electronic devices. The semiconductor industry is subject to a continuous evolution, but it has to face several challenges, including rising costs, increasing competition, and the need to always develop new and innovative products. On the other hand, implementation of Industry 4.0 technologies is often complex and costly, requiring high investments and new processes and systems (see [2] for a recent discussion).

Producing semiconductors is a highly complex process. Downtime is a major problem, causing billions in profit losses. Even a relatively small breakdown can interrupt production and oblige that the process be restarted from the beginning. This is a major drawback, considering that currently production is scheduled on a 24/7 basis. In addition, in many cases production is shared between factories located far away each other (even in different countries), which increases the time required to resolve the issues that occur. Finally, one has also to consider complications arising by inevitable rare events in the “black swan” class such as the COVID-19 pandemic, which restrict movements—they are random conditions whose impact should be reduced as much as possible.

Some best practices to make fabrication processes of semiconductors more efficient integrate Virtual Metrology tools [3,4,5,6,7] and multiscale process simulation modules (Virtual Design of Experiments, V-DoE) [8,9,10,11] at each step of the whole manufacturing flow.

In this respect, Machine Learning (ML) and, more recently, AI are key tools for Industry 4.0 in semiconductor production because they can control and monitor the manufacturing process at most stages (e.g., lithography, etching, doping). They can analyze sensor data in real time and predict potential issues or inefficiencies. In practice, they are able to improve process control, increase efficiency, and reduce downtime. See the review in [12] for general methodologies, and particularly, [13] for ML and [14] for AI.

Besides manufacturing optimization and control, another important area of intervention of Industry 4.0 is Predictive Maintenance (PdM), where suitable machine-dependent software analyzes sensor data gathered from the hardware equipment and tools during a process, and predicts when such equipment requires maintenance, before a breakdown occurs, avoiding unnecessary maintenance and rupture of hardware tools as well. See [15] for an up-to-date history in industry with its growing importance and [16] for its impact in the semiconductor industry specifically, also based on interviews.

Indeed, PdM proved to be successful in most types of industry (metallurgic [17], aircraft [18,19], textile [20], civil infrastructure [21], pharmaceutical [22], vehicles [23], batteries [18], steel [24,25], and logistics [26], to give some examples).

The high complexity of the semiconductor production system is not compatible with a unified framework able to face all related PdM problems. However, some categorizations are attempted in the literature aimed at defining groups of homogeneous frameworks from the system point of view [18,27,28,29,30,31,32,33,34,35].

As far as methodologies and related software are concerned, a categorization is attempted in [36] (with a specific section for semiconductors, as in [37]), in [38,39,40,41] for ML particularly, and [42] for graph-based approaches.

Due to the high heterogeneity of processes in semiconductor fabrication, a large variety of PdM frameworks and endowed methodologies are used. In practice, each application of the semiconductor industry together with its endowed methodology for PdM can be considered as a case study. PdM has been applied, e.g., to transport of wafers [43,44], prediction of wafer failures [27,37,45,46,47,48], ion-beam etching [49,50], supply chain [51], vibration during normal operation [44], and pump and abatement equipment [35].

The present paper considers a case study of PdM in the semiconductor industry, namely, the mechanism of holding wafers inside a growth chamber through four Pins. Such Pins are subject to degradation upon their use, and their rupture during normal operations implies loss of money in terms of wasted wafer and downtime. Before this study, Pins were replaced in the case of their actual failure or every six months, configuring a Preventive Maintenance (PM) scheme. The aim of the present study is to develop a prototype of a monitoring system based on optical sensors endowed with an image processing software able to predict possible failure of the Pin. In this way, unnecessary replacements of the Pins are avoided, so that downtime is reduced. In practice, one moves to a PdM approach.

The paper is organized according to the following scheme. Section 2 introduces the problem from the engineering point of view, and particularly the hardware part of the adopted solution based on an optical sensor. The related image processing system is discussed in Section 3, including the indicators introduced for monitoring the system in Section 3.3 and Section 3.4. Section 4 presents the data set gathered in two years of study. The main results of the study are presented in Section 5, also introducing the app PINapp, which was developed to monitor the Pins and to signal potential conditions of abnormality. Finally, a discussion is drawn in Section 6.

## 2. The Problem

**Figure 1:**
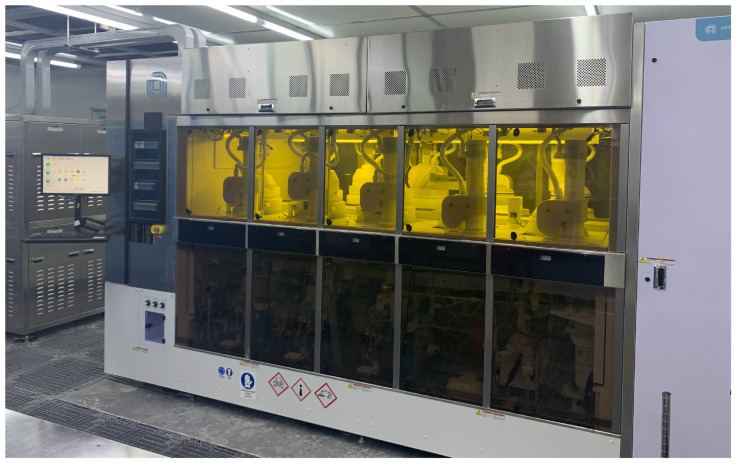
Copper electroplating deposition equipment (STMicroelectronics, Catania Wafer Fab Operations, Catania, Italy). Source: STMicroelectronics.

The fabrication of integrated circuits is a process by which devices are grown on a wafer to make the required final product. It is a sequential procedure of applying ordered processes to deposit new layers of microelectronic features on the wafer. The number of procedures can be as high as some hundreds (depending on the final product) and they occur over a time period of weeks, during which the wafers are transported from their rest position to an electrochemical deposition system where the growth process occurs. This system is an enabling tool for the production line and it is composed of six process chambers (Figure 1).

**Figure 2:**
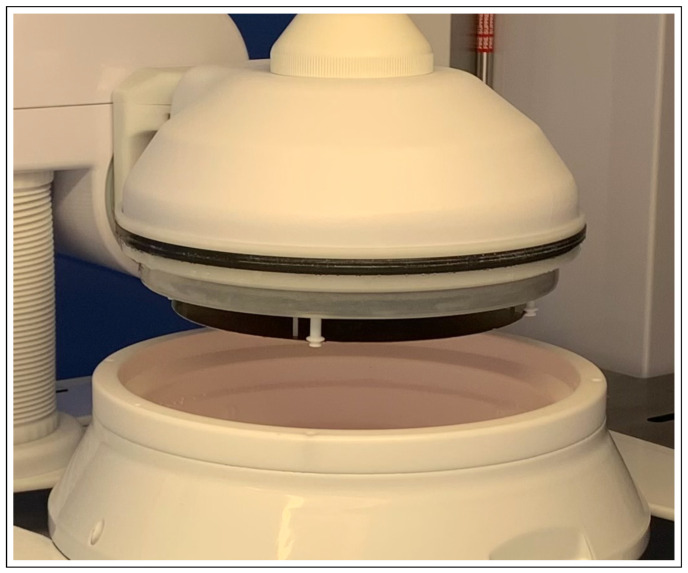
Detail of the Electrodeposition chamber, showing the mechanism that holds wafers by means of the four Pins. Source: STMicroelectronics.

Inside each chamber, the wafer is held by four Pins located approximately equispaced on the boundary of the wafer (Figure 2).

The Pins can assume two states:

- Open, which allows the transportation of the wafer from and to the chamber;
- Closed, which blocks the wafer for its growth processing.

These states are triggered by a piezoelectric transducer at the start and end of the growth process inside the chamber. Such Pins are normally subject to ruptures due to several reasons in principle (e.g., stress caused by the continuous switch between opening and closing conditions). Therefore, they undergo a PM task. Before the study, this was scheduled at fixed periodic times, and was not supported by indicators of real conditions of the Pins. This approach can lead to two extreme situations:

1. The Pin degradation occurs before the scheduled replacement time. In this situation, severe damage to wafers and chamber occurs, as corrosive chemicals are used during the process. As a consequence, the wafer is destroyed and has to be wasted, and the growth process has to be started again from the beginning. In addition, the production line is interrupted to allow cleanup of the chamber and its calibration, with the effect of an increased downtime of the system. Both consequences increase the cost of production. At the beginning of the project no alert or warning was present to avoid this circumstance.
2. The Pins are replaced even if they are still in good condition. This second case brings an extra cost of spare parts and labor because the estimated lifetime of Pins can vary because of many factors, such as quality of elastomer materials and number of wafers processed during the interval between replacements.

The objective of this work is to develop an integrated intelligent solution to support the maintenance, reducing the number of scrapped wafers and the cost of maintenance through less part replacement and labor.

In the ideal case, the system is thought of as a network of camera sensors that gather images of the Pins at regular times (e.g., daily) during normal operations. It is endowed with an image processing software that, starting from the images, alerts in case conditions occur that can lead to a rupture of the Pins.

Before the project, the replacement of the Pins occurred every six months on the basis of a PM scheme following a Run to Fail program. In addition, a schedule was applied with a check every six months to verify the correct clamping of the wafers and to replace the counter springs that induce the movement of the Pins. Very little was known about the final actual causes of the rupture and how the rupture process occurs. The main reason is that due to the high cost of a rupture of the Pin during the normal process (e.g., waste of the wafer, cleaning up, and reinitialization of the chamber), a conservative schedule of replacement is preferred, with the drawback of probably unnecessary replacements and consequently of frequent downtime of the production.

In practice, it was not clear at the beginning of the project which type of artifacts to expect that give rise to the rupture of the Pins, e.g., whether mechanical due to the stress of the Pin movement triggered by a piezoelectric device, or chemical due to the interaction with materials present in the chamber during normal operations. In addition, it was not clear whether the rupture occurs suddenly or at the end of a short or long period of degradation. Nor it was clear if to expect deformations of the main Pin structure, change of color due to the stress acting on some specific parts of the plastic of the Pin, or local artifacts (e.g., holes or cuts) occurring in specific positions of the Pins. Finally, it has to be mentioned that it was not even known in the beginning which type of artifacts could have been present due to normal operations in the chamber that were not prone to a rupture of the Pins.

For this reason, the ideal configuration of the system is thought of as a network of four cameras dedicated to the four Pins with sufficient spatial resolution, endowed with an image processing software that is pixel-based and able to detect artifacts even at little spatial resolution. As a secondary step, artifacts detected by the image processing system have to be interpreted, with the objective of discriminating between the ones normally occurring during the process and the ones potentially giving rise to a rupture of the Pin.

This approach transforms a PM approach into a Predictive one, triggered by the detection of anomalies prone to a rupture of the Pin.

**Figure 3:**
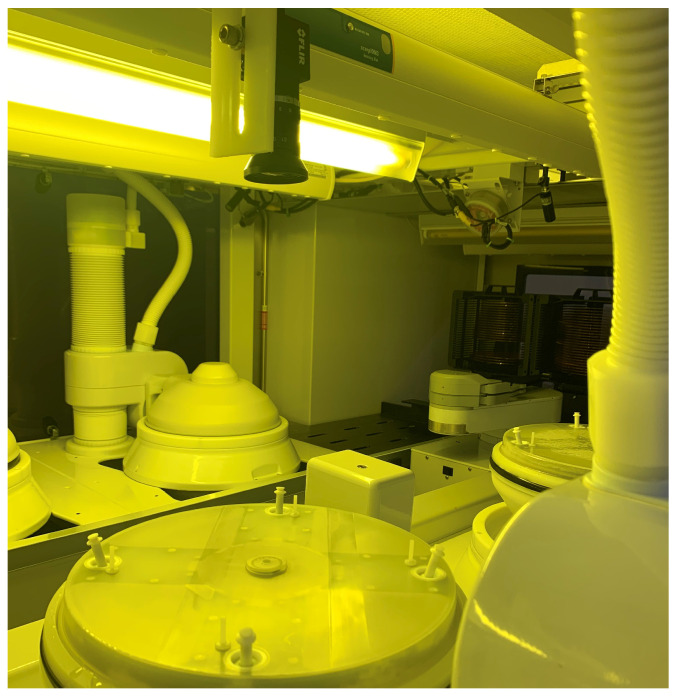
Image of the camera at the top that captures the 4 Pins of the chamber below.

For practical and technical reasons the project has been realized with a prototype simplified camera system based on one camera that is centrally located, simultaneously viewing all four Pins of the chamber (Figure 3).

### 2.1. The Camera System

Camera resolution (5MP) corresponds to a pixel resolution of about 0.1 mm at the distance of the Pins from the camera. Experience showed that this is enough to detect some artifacts, even strictly local, which are of the order of a few pixels. However, such artifacts are generally not in the same positions, so it is important to track well their presence over time.

The camera is also able to take videos at a frame rate of 24 fps. In principle, they could be recorded during the opening/closure of the Pins and reveal possible anomalies. However, the entire movement occurs in a very short time and completes in two frames (less than 100 ms), so videos did not reveal useful information to detect anomalies (view the Video S1 in the Supplementary Materials for an example).

The main specifications of the camera are shown in Table 1.

The camera is assembled on the top of the chamber (Figure 3), approximately at the center of the wafer so to equally cover most of the frontal part of all four Pins (the ones that hold the wafer). Unfortunately, the distance between the camera and the four Pins is not the same; therefore, a compromise had to be found to make all Pins as least defocused as possible. As a consequence, all Pins are somewhat defocused in the image.

## 3. Image Processing System

As mentioned, without an accurate knowledge of possible situations prone to a rupture of the Pins, a strategy was developed for detecting possible anomalies in an image at a high spatial resolution, in order to pick up even small anomalies. For this reason, the methodology will mainly work pixel-wise. In practice, the system will gather images of a wafer with the four holding Pins at a regular as possible frequency; starting from this sequence of images, the objective is to identify possible anomalies on a pixel basis. For this reason, one needs the images to keep as similar as possible with time, both from the spatial and lighting points of view, so to avoid the risk of interpreting variations due to changes of position or lighting as artifacts as much as possible. Actually, normal operations on the chamber due to ordinary or extraordinary maintenance can slightly change its position relative to the camera and/or its lighting conditions, thus generating new anomalies due to these changes and affecting the capability of detecting true outliers. Such operations sometimes required the camera to be unmounted and then mounted back, with the net result that its position was not exactly the same as before. As shown later, variations in the position of the Pins with respect to the camera as high as above 100 pixels (which correspond to more than 1 cm) were observed. Therefore, images underwent a pre-processing aimed at keeping the Pins in the same position and lighting conditions. This pre-processing consists of the registration and Equalization of images.

### 3.1. Registration

A key aspect of the methodology to avoid the detection of artifacts not related to Pin degradation, also considering the high spatial resolution needed to detect small artifacts, is to keep the Pins fixed at the same position over the images in time. To overcome the small variations consequent to the normal processing and, especially, to scheduled and unscheduled maintenance, a registration algorithm has been applied to the images. To this purpose a reference image has been chosen, and the entire set has been registered with respect to that image.

Let $I^{k}\equiv I^{k}\left(x,y\right)$ be the gray level of the *k*-th image, with (x,y) the coordinates of the image. When (x,y) coincide with a pixel of the image, the notation $I_{ij}^{k}\equiv I^{k}\left(x_{i},y_{j}\right)$, i=1,…,M, j=1,…,N, will be directly used, with *M* and *N* being the number of pixels of the images to be registered on the horizontal and vertical coordinates, respectively.

The reference image ($I^{\bar{\ell }}$) has been chosen initially as the one among the first ten gathered images that shows the least variation with respect to the other ones. Variation, $\bar{\ell }$, is measured by Equation (1)

(1) ℓ¯=arg mink=1,…,10∑ℓ=1ℓ≠k10∑i=1M∑j=1NIijℓ−Iijk2.

Considering the structure of the chamber system, a rigid transform is searched that best matches each image to the reference one. To this purpose, a methodology based on affine transformation is adopted [52]. Here, a transformation of a 2D image is represented through a 2 × 3 transform matrix by Equation (2)

(2) $$\left|\begin{array}{c} x^{\prime }\\ y^{\prime } \end{array}\right|=\left|\begin{array}{ccc} a_{11} & a_{12} & d_{x}\\ a_{21} & a_{22} & d_{y} \end{array}\right|\left|\begin{array}{c} x\\ y\\ 1 \end{array}\right|,$$

with (x,y) being original coordinates of a pixel, $\left(x^{\prime },y^{\prime }\right)$, the transformed ones and $T\equiv \{a_{11},\ldots ,a_{22},d_{x},d_{y}\}$, representing the set of parameters of transformation. $d_{x}$ and $d_{y}$ are responsible for the shift of the image in the horizontal and vertical direction, respectively; parameters $a_{11},\ldots ,a_{22}$ induce several types of transformations of the image. For example, $a_{11}=\cos\theta$, $a_{12}=-\sin\theta$, $a_{21}=\sin\theta$, and $a_{22}=-\cos\theta$ generate a rotation of the image by angle θ. In the present work, to take into account all possible movements of the chamber relative to the camera including tilt, besides translations and rotations, all the six free parameters of the affine transformation will be considered, therefore, including also shear and scale.

In summary:

- One first chooses a reference image with which to coregister all the other ones using the procedure described above (see Equation (1));
- Then, each image is coregistered with the selected reference image choosing the best parameters $\left\{a_{11},\ldots ,a_{22},d_{x},d_{y}\right\}$ of the transformation (2).

Step (b) is accomplished using Least Squares through the cost function of Equation (3), which is similar to Equation (1)

(3) $$\min_{T}\sum_{i=1}^{M}\sum_{j=1}^{N}{\left(I^{k}\left(x^{\prime },y^{\prime }\right)-I^{\ell }\left(x_{i},y_{j}\right)\right)}^{2},$$

with $x^{\prime },y^{\prime }$ being transformed coordinates of the pixels $\left(x_{i},y_{j}\right)$ according to Equation (2). Since in general $\left(x^{\prime },y^{\prime }\right)$ do not coincide with a gridpoint of the image, then a simple bilinear interpolation is considered for estimating the value of gray level in the transformed point based on the four closest gridpoints.

In practice, in some circumstances related to severe maintenance, the camera has to be disassembled and then reassembled; therefore, relatively high displacements of the camera occur. Therefore, the coregistration process goes through two steps: in the first one only optimal displacements restricted to gridpoints are sought through an exhaustive search up to a maximum displacement (200 pixels). This operation does not require interpolation of images and therefore is computationally fast. Then, the full affine transformation accounting for simultaneous translations, rotations, and shear is applied considering all the six free parameters. In addition, transformation is sought separately for each Pin, considering a small subregion of the image around the Pin, enlarged to take account of transformations of the image. This step is important because some maintenance operations (e.g., replacement of the counter springs) are applied to the Pins separately, and this could produce minor independent movements of the Pins. In addition, working on the separate Pins has the advantage of speeding up computations.

Alternative approaches to coregistration could be possible, e.g., based on the Fourier Transform [53].

### 3.2. Lighting Variation

**Figure 4:**
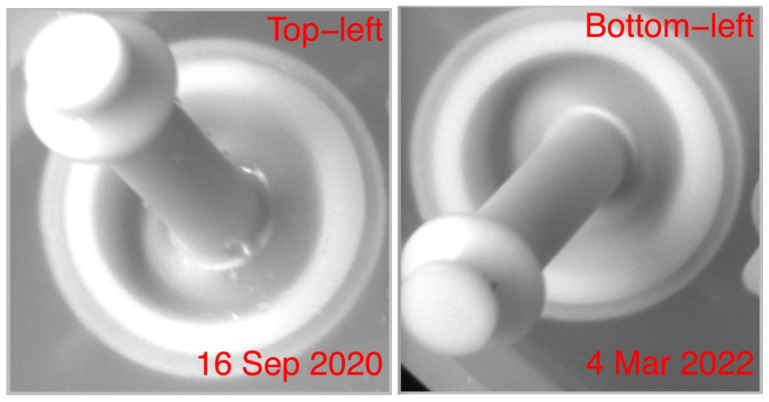
Example of artifacts due to normal processing inside the growth chamber: drops of liquid at the base of the Pin (**left**) and dirty particles under the top part of the Pin (**right**). Source: own research.

Even though the camera was in constant light conditions with time, minor variations in lighting inside chambers may occasionally or systematically occur. Therefore, to clean gray levels from the effect of varying lighting conditions, a methodology was applied to keep the overall color structure of the image as constant as possible and independent of the external variations. In the literature, there exist several methodologies to this purpose. One of them falls in the framework of Equalization, which aims at making a gray level histogram as constant as possible. As a by-product, such methodologies also increase the dynamic range of the images to have a clearer representation of the images, at least from a visual point of view. Equalization was performed using the Contrast Limited Adaptive Histogram Equalization (CLAHE [54]). However, a drawback of such methodologies is lack of saturation for many images. Actually, images show well-defined parts that are saturated (white color, that is gray level 255; see, for example, the top or basis of the Pins in Figure 4). After Equalization, in many cases the saturation level was reached for a slightly lower gray level. The difference gave rise to bimodal distributions of gray level and therefore to artifacts in picking outliers due to Equalization rather than to images themselves. Therefore, the Equalization methodology by CLAHE, which processes each image separately, has been replaced by a Histogram Matching one [52] (see [55] for a recent review). Unlike Equalization, Histogram Matching adjusts the histogram of an image to match the histogram of a reference image. Indeed, Equalization is a particular case of Histogram Matching, with the reference histogram being constant. Using Histogram Matching and a suitable choice of the reference image, a saturated zone with gray level 255 remains at the same level after processing. This procedure requires a reference image with which to match all other ones. This has been accomplished by searching for each Pin in the image among the first 100 (when available) that has the least variability with respect to all the other ones.

It has to be remarked that registration can also affect Histogram Matching: the shifts/rotations observed over time also change the region of the image to equalize, with the consequence of slightly biasing Equalization. Therefore, Histogram Matching was applied twice (before and after registration).

### 3.3. Outlier Indicator

The main step of the image processing framework is to detect possible anomalies, which are indicators of an incoming rupture of the Pin. As mentioned above, the lack of knowledge of typologies of anomalies required investigation from a high spatial resolution that is pixel-based.

The objective is reached by analyzing for each pixel the distribution of gray level of images over time. Several elements contribute to such distribution: first of all, the natural variability of gray levels due to random fluctuations of the system over time (e.g., micromovements); then, effects of the registration and Equalization processes, due to not perfectly accurate reconstruction and/or interpolation error; more importantly, the presence of physical artifacts on the Pins, which can be normally produced during the growth process inside chambers or related to failures on the Pins that will cause their rupture; finally, variations eventually arising from a possible deformation of the Pins due to mechanical stress have to be mentioned, which could involve an entire region of pixels.

A specific category of variations, related to the camera system, is the focus of the images. As mentioned in Section 2.1, the prototype system installed in the project is given by a unique camera that covers the four Pins. Therefore, none of the Pins are properly focused, which results in a typical blurring effect of the images. From the gray level point of view, this means that a specific anomaly condition in a pixel (e.g., gray level significantly higher or lower than normal conditions) spreads out to nearby pixels, in the sense that the anomaly of gray level in the involved pixel is reduced (lower or higher gray level, respectively), while it is increased (higher or lower gray levels, respectively) in nearby pixels.

All these elements result in different and intricate distributions of the gray level according to the different situations. As a rough categorization, from the mathematical point of view, one can observe the following:

1. Unimodal distributions, typically representative of no artifacts, with variability given by natural conditions during operations and/or error of registration and/or Equalization and blurring from the camera being out-of-focus;
2. As a particular case, saturated pixels, where the gray level is always at its highest value 255 (white), absorbing natural, camera, and image processing variations;
3. Bimodal distributions, due to two different conditions over time (e.g., normal operations and artifact present); in general both conditions are highly unbalanced;
4. As a particular case, widespread distributions, typically arising from normal conditions and artifacts that are spread along nearby pixels mainly due to camera defocusing.

**Figure 5:**
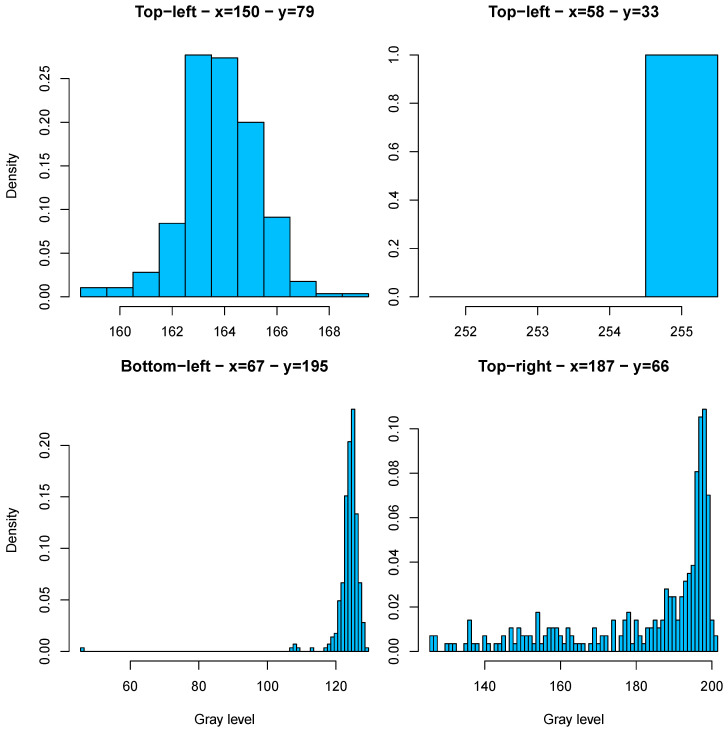
Sample distributions of gray levels in selected pixels: unimodal distribution (**top left subplot**), saturation distribution (**top right subplot**), bimodal distribution with predominant normal state (**bottom left subplot**), and widespread (**bottom right subplot**). The Pin and coordinates of the pixels are shown for each subplot. Source: own research.

Examples of such distributions are shown in Figure 5.

Considering this variability of distributions, in particular bimodal ones, an anomaly indicator has been considered based on percentiles rather than standard deviations more suitable for Gaussian-like distributions. In addition, to take account of the skewness of some typical distributions, the indicator will separately take into account both the left and right tails of the distributions. Therefore, this indicator, O, is defined in Equation (4)

(4) $$O\left(I\right)=\left\{\begin{array}{cc} \frac{I-I_{50}}{2(I_{75}-I_{50})}, & \mathrm{if}\;I\geq I_{50};\\ \frac{I_{50}-I}{2(I_{50}-I_{25})}, & \mathrm{if}\;I<I_{50}, \end{array}\right.$$

where $I_{p}$ indicates the *p*-th percentile of the distribution of gray level *I*, and $I_{50}$ is the median. This indicator does not rely on the InterQuartile Range (IQR) that is more suitable for symmetric distributions.

In other words, the outlier indicator O expresses the distance of the gray level from the median relative to the interquartile range estimated on the side of the distribution (left or right of the median) where the gray level lies.

Note that for symmetric distributions this indicator is asymptotically equivalent to using the usual IQR (therefore not two-sided), and a value O>1.5 corresponds to the usual rule-of-thumb of detecting an outlier. See [56] for the methodology and some alternative more elaborated indicators. In this project, the threshold (usually 1.5) to decide whether a gray level is an outlier can be tuned through a handle by the process engineer to have a better feeling for the status of the Pins.

Finally, it has to be remarked that the detection of outliers is based on the basic assumption that there exists a normal, majority status, where the gray level assumes most of its own values, and an abnormal (anomaly) minority condition where the gray level assumes exceptional values. In other words, the anomaly has to be present for a minority of the entire time sequence.

At the end, each pixel of each image has its own associated percentile value and outlier status indicator.

### 3.4. Related Indicators

Outlier Index O is the key indicator to detect an anomaly at the pixel level. It has been chosen because, in the beginning of the project, there were almost no indications of the type of anomaly to expect that could cause the rupture and failure of the Pin. Lessons learned by processing images over time have suggested that there are several types of anomalies, mostly due to the normal process and not related to a failure of the Pin. They include drops of liquid that are mostly present on the base and on the middle part of the Pin, shifts of parts of the system external to the Pin, and dirty particles that attach to the Pin, especially just under the top part of the Pin. Each of them has its own characteristics in terms of gray level distribution: for example, dirty particles show a lower gray level (darker color) than normal values, while drops of liquid affect the distribution of gray level through a much larger variance with respect to the normal state. Examples of artifacts are shown in Figure 4.

In order to distinguish normal artifacts from conditions that could cause a failure of the Pin, the starting observation is that normal artifacts are limited in time, whereas artifacts that can cause the failure are permanent. Experience on the images gathered so far revealed that drops of liquid last for a few days at most, whereas particles of impurities apparently lasted up to about 1 month.

It is not possible to give a more accurate estimate of the duration of these artifacts because images were not regularly collected every day. Therefore, the somewhat strong but necessary assumption has been made that an artifact present in a pixel in two consecutive days of measurement is also present in the middle days, even though no images have been taken. Based on these arguments, three new indicators have been developed:

- Occurrence of Outliers. This is defined as the fraction of days in a certain period in which the gray level in a pixel is in a condition of outlier. This includes outliers lasting only one day and, for the arguments above, days between two consecutively detected outliers;
- Persistence of Outliers. This is defined as the fraction of subperiods in a certain period of time in which the gray level is continuously in a status of outlier. Actually, both Occurrence and Persistence indicators are similar—essentially, they differ only in isolated pixels that are not counted for the latter. However, when images are taken every day with regularity, so that the Persistence is exactly estimated, this estimator will significantly differ from the Occurrence one and is expected to provide better indications. It has to be remarked that bad quality of images, in particular lack of focus, can also affect Persistence, in that, for example, a dark impurity is spread over adjacent pixels showing higher values of gray level (lighter gray) up to possibly misdetection of the outlier condition;
- Mixture of distributions. This indicator arises from a simplistic model where the distribution of gray level is approximated as a mixture of two Gaussians with their own mean and variance (see [57] for a comprehensive treatment of Gaussian Mixture Models, GMMs). Such components represent the normal and artifact states. This is a different representation of an outlier than the robust one based on median and IQR. GMMs are solved using the Expectation–Minimization algorithm (EM, [58]). Several software packages are available to estimate the related parameters (mean of the distributions, covariance, and weights); however, they are computationally intensive for our application, considering that such components have to be found pixel-wise. An alternative is to rely on a faster clustering algorithm, that naturally sorts data into two groups (clusters). *k*-means (also known as Lloyd’s algorithm) is one of the most popular methods. Both EM and *k*-means have many similarities and, even though they are not equivalent, are often used interchangeably [59]. *k*-means assigns the gray level of a pixel at each date to one of two clusters according to the lowest distance from the centroid of the clusters in an iterative way. After sorting the gray levels, mean and variance of each cluster (components in the GMM notation) are easily computed from the data within the clusters. The proportion between the two components is estimated by the frequency of data in the two clusters. Finally, since the attribution of the class index 1 or 2 is random, we define as an outlier cluster the less numerous by the very definition of outlier. Of course, in the case of permanent artifacts that do not disappear, in the long term, the outlier cluster tends to become primary.

### 3.5. Filtering

Despite the accurate pre-processing of images by registration and Equalization, false positives (i.e., normal conditions estimated as anomalies) are possible. This is due to several reasons: first of all the natural variability of the system due to the industrial process statistically widens the range of the levels of gray when the time series of images becomes longer. In addition, registration and Equalization, yet accurate, are not perfect.

The main consequence of these factors is the presence of outliers at isolated pixels (the so called salt-and-pepper effect). To mitigate this problem, the assumption has been made that an anomaly cannot be locally isolated, but it has to be surrounded by at least a certain number of pixels *F* also showing an anomaly. Due to the high spatial resolution of the camera, the anomaly detection process will not practically miss the real ones also considering that most artifacts (e.g., drops of liquid) are spread over several pixels, and the smallest ones (particles) involve a few adjacent pixels. Therefore, anomalies will be discarded that are not surrounded by at least *F* anomalies. In case F=8, the entire box around a pixel will be considered.

## 4. Data Set of Images

The data set gathered by STMicroelectronics is composed of images gathered at uneven time intervals (one per day at most). For each day where images are available, two different images are taken, corresponding to a situation of open and closed Pin. The former allows the release of the wafer to be moved to its rest position, and the image is taken when the camera is idle, before the growth process starts inside the chamber; the latter blocks the wafer inside the chamber for its treatment with the growth of the semiconductor, and the image is taken at the end of the process, again when the camera is idle. Each image is provided in bitmap (BMP) format, uncompressed, black and white, having a pixel resolution 2048 × 2448 (5 MB, see Table 1). At the end, the data set is composed of 285 images at disposal for each state, dated from 25 August 2020 to 9 August 2022, with the monthly frequency shown in Table 2.

On average, 11.5 images are available each month, with a highly variable range from 10% availability in December 2020 to a maximum in September 2020 (67%). The average time between images is 2.5 days. For almost one-half of cases images are gathered on consecutive days, while for almost two-thirds of cases the time interval between images is up to two days. However, for almost 3% of cases the time distance between images is from ten days and up, with a maximum of 28 days between 7 December 2021 and 4 January 2022.

**Figure 6:**
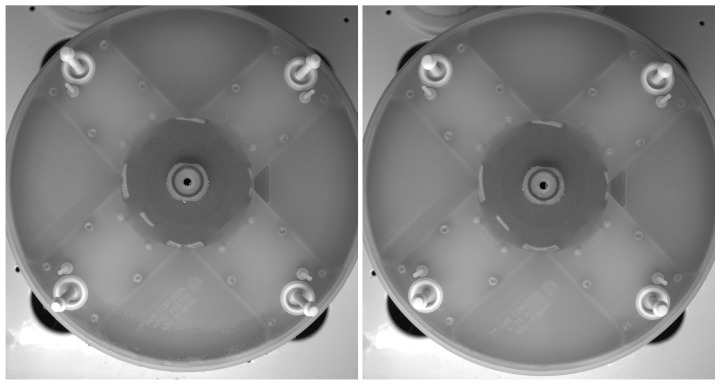
Sample image taken for the camera in open (**left**) and closed (**right**) condition of the Pins. Source: own research.

Sample images in both open and closed conditions of the Pins are provided in Figure 6.

Comparing images of open and closed Pins, it is possible to notice that the visibility of Pins in the former case is much better then the latter one, to the point that images of Closed Pins can be considered completely redundant and do not add further information. Therefore, they have been discarded and analysis will be made only on images of open Pins.

## 5. Results

The main result of this study is understanding the main mechanism that leads to the rupture of a Pin. During normal operations, the Pins are quickly moved towards and back from the wafer to hold it, so that their upper part comes in tight contact with the external boundary of the wafer. Such continuous movement produces a rubbing of this part of the Pin, with the consequence that its friction increases. Therefore, dirty particles that are produced during normal growth operations on the the wafer are not completely washed out from the cleaning steps, but they can remain locally attached to the Pin for some time with a probability that increases with the friction of the surface. In the long term, as soon as the degradation process progresses, rubbing provokes a local thinning of the Pin surface. Thus, besides the increased friction, in the end a small hole or cut will be produced with the consequence of the rupture of the Pin and interruption of the growth process on the wafer, if the Pin is not replaced on time. Incidentally, the hole is visually indistinguishable from dirty particles, both being of very dark color.

Video sequences of the images gathered along the entire period of study are included in the Supplementary Materials in Videos S2–S5 for the Bottom left, Bottom right, Top left, and Top right Pins, respectively.

The final result is that when degradation of the Pin progresses, there is a greater presence of visible dirty particles, with an increasing concentration and a higher persistence. In the chamber of the present study, this effect is clearly visible on one Pin, but not to the point of its rupture (that is, production of the hole/cut), that indeed did not occur until many months after. The rupture of the Pins was observed in other chambers where the camera system was not available. This proves that the degradation process of the Pin is slow.

An application software (PINapp) was developed to monitor the chamber and the Pins remotely, also providing alert information in a visual graphic form. The entire analysis of PINapp is applied to the images obtained after the image processing chain described in Section 3 (registration of images, reduction of lighting variation). Video sequences of the images gathered along the entire period of study showed above display the good behavior of the registration and lighting correction.

**Figure 7:**
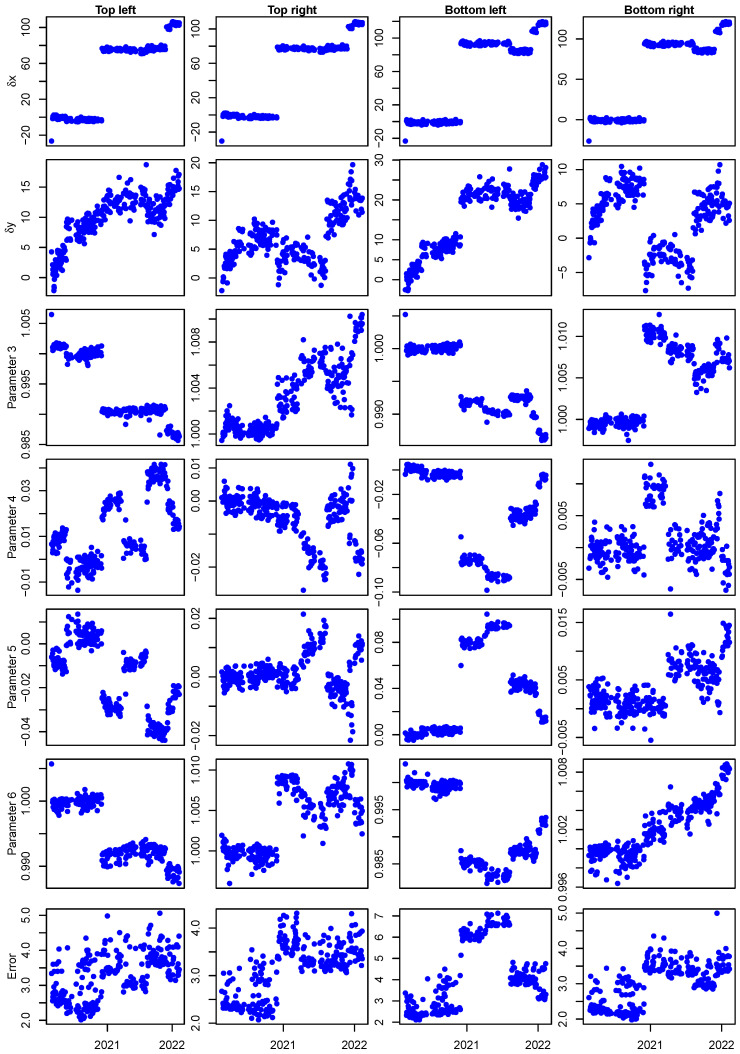
Registration parameters *T* (Equation (1)) for the four Pins (columns) and the six registration parameters of the transformation (first six rows: $\delta_{x}$, $\delta_{y}$, in pixels, $a_{11}$, $a_{12}$, $a_{21}$, $a_{22}$, respectively). The last row reports the registration error according to Equation (3). Source: own research.

Figure 7 shows the time series of the six registration parameters *T* of Equation (1) discussed in Section 3 for the four Pins over the entire period of study ($\delta_{x}$, $\delta_{y}$, in pixels, $a_{11}$, $a_{12}$, $a_{21}$, $a_{22}$, respectively). An estimate of the registration error by Equation (3) is also reported.

Figure 7 shows that during the 2-year period of the study, the system underwent several adjustments due to scheduled and unscheduled maintenance that modified the position of the camera over time. In particular, a total shift in the horizontal position can be observed of 120 pixels, which corresponds to more than 1.2 cm. The shift in the vertical position is limited to about 20 pixels maximum (more than 0.2 cm). Despite the relatively large movements of the camera system, registration root mean square error kept limited to 5 gray levels for all Pins and 7 for the Bottom left one (Figure 7). Please note that this error also includes lighting variation.

### 5.1. Application PINapp

The aim of PINapp is twofold:

1. To monitor the time evolution of the chamber and of the four Pins in order to visually check predicted potential anomalies;
2. To provide indicators of potential anomalies in a graphical form, endowed with graphical tools for supporting indications.

PINapp is developed within the R environment using the Shiny package; therefore, it is highly interactive and meets the requirements of the industry for a highly user friendly interface. It runs on all platforms (Windows, Linux, Mac). It can run on a local machine or through a client–server architecture, with more users simultaneously connected to a server remotely. PINapp works on an intermediate data set obtained from the original images through a suitable software that runs image pre-processing and computes the Outlier index.

**Figure 8:**
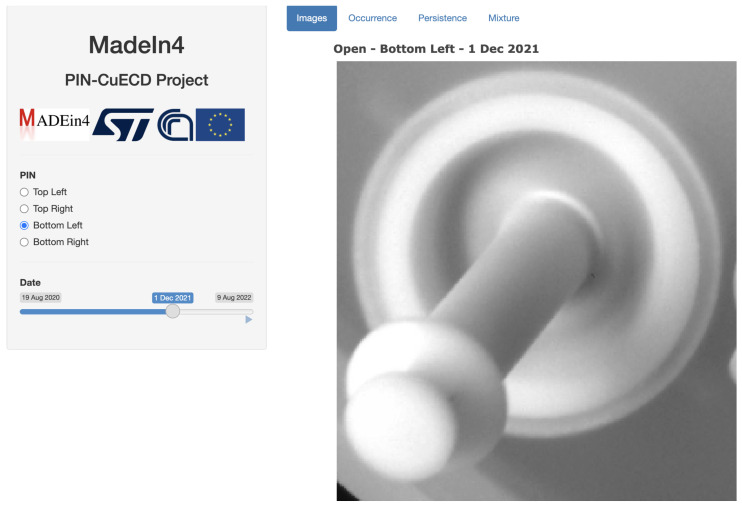
Visualization of images in PINapp through the tab Images. Source: own research.

**Figure 9:**
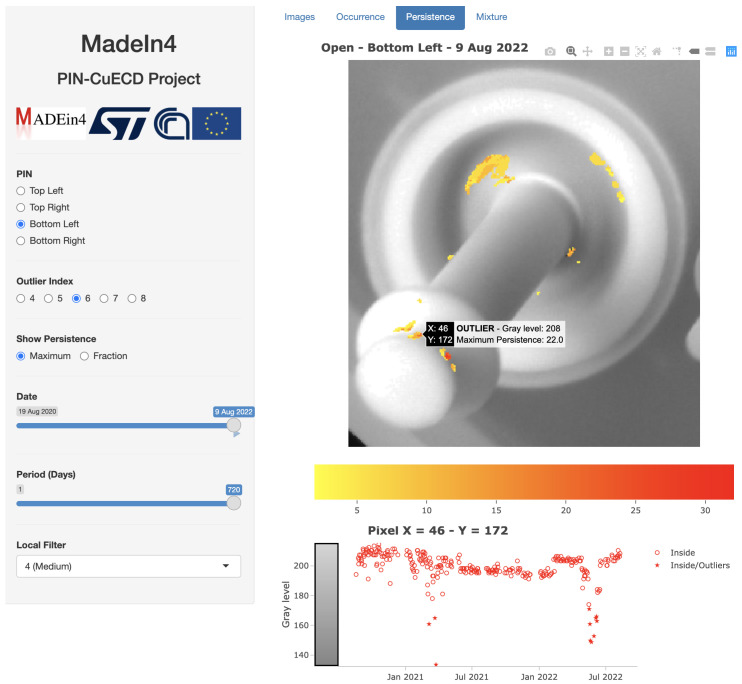
Map of Persistence through the tab Persistence. Map refers to the Bottom left Pin for the entire duration of the study (2 years) and values of the Outlier index O≥6, while underlying image refers to the last day of the study. Menu for the choice of user parameters is on the left. The time series of the gray level of a pixel selected by cicking on a corresponding location with the mouse is shown in the bottom right. Detailed information on a pixel is also shown in correspondence to the pixel (in the example coordinates x=46, y=172). Source: own research.

The visual interface has two windows (see, e.g., Figure 8). The right one includes figures and plots to visualize images of the Pins and information on artifacts. Its actual content depends on the selection by the user of a tab in the upper part among Images, Occurrence, Persistence, and Mixture, that mostly reflect the indicators developed in Section 3.3. The left window is dedicated to parameters that a user can tune for the visualization of the right window. Such parameters depend on the chosen tab, but always include the choice of the Pin (parameter PIN, among the four available, Top left, Top right, Bottom left, Bottom right) and the date (Date) at which to show the image shown in the right window. In addition to and depending on the chosen tab, other tuning parameters can be present (see, e.g., Figure 9) as the number of days when to restrict visualization (hlPeriod (days)) up to the date of the image shown as selected above, the value of the minimum Outlier index O (Equation (4), hlOutlier Index) for the map of Outliers, and the amount of filtering applied to the maps (Local Filter).

This Section will describe only the most interesting features, with the purpose of showing the main results of the present study applied to the data gathered (see Section 4).

### 5.2. Time Evolution of Pins

Monitoring evolution of the Pins is accomplished through the tab Images of PINapp (see Figure 8).

The selection of parameters is made on the left side of the graphical window. The Pin can be selected using a suitable radio button, and the date of the image through a cursor. It is also possible to generate an animation of the video sequence.

### 5.3. Persistence of Outliers

Outliers (O) have been defined in Section 3.3 and indicate whether the gray level of a pixel in an image on a certain day can be considered regular or at the tail of its statistical distribution (outlier). Section 3.4 already mentioned that an outlier is not necessarily an indicator of a possible failure of a Pin, but most times it is associated to artifacts that normally appear in the processing of a wafer (e.g., droplets, dirty particles). Therefore, in order to help distinguishing artifacts potentially prone to a failure from the regular ones, an indicator has been developed, Persistence, which measures how long an artifact is present on a Pin (Section 3.4). This indicator is graphically shown in a proper window of PINapp, recallable by the tab Persistence. An example is shown in Figure 9.

The graphical window of Figure 9 refers to the Bottom left Pin and shows the map of the outliers detected along the entire period of study (720 days) until the last date (9 August 2022, whose image is shown in that Figure), with value O≥6, and color bar provided below the image of the Pin. The graphical window provides further useful information. Below the color bar, a time series plot is shown of the gray level in one pixel of the image (gray color also shown on the left axis). Time series points are indicated by a star symbol in the case that they refer to outliers, and by a circle otherwise. In addition, they are represented as a red color in the case that they are within the time window selected in the left menu and as a blue color otherwise (in the example of Figure 9 the time window covers the entire time span of the study so that all points are within the time series and are therefore in red color). The pixel for which the time series is shown is selected by clicking on the corresponding position in the image. By hovering on the pixels with the mouse, further detailed information on the underlying pixels are shown (see Figure 9), namely, coordinates of the pixel, grey level at the date set in the left menu, and, in the case that the pixel is selected to be shown by the color map because of significant Persistence in the considered period, also indication that that pixel had Outlier values in the considered period and the value of Persistence in the selected time window.

There are three main locations of outliers shown in the color map: at the base and on the middle part of the Pin (indication of the presence of droplets); on the top part of the Pin; and externally to the Pin. The interesting artifacts are on the top part of the Pin, because it is in contact with the wafer and therefore subject to rubbing. Such artifacts are mostly the effect of dirty particles that adhere to the Pin, which are part of the normal process of growth of the wafers and therefore not related to a potential damage of the Pin in principle. However, experience during the study and a direct visual check on the Pins showed that it is the place where failures of the Pin could be generated, essentially in the form of small holes or cuts that cannot be visually distinguished from dirty particles. In this respect, the time series plot of gray level in a pixel helps in understanding when the artifact occurs along the time span considered and for how long. In Figure 9, for example at the pixel of coordinates x=46, y=172, artifacts occur on 6–25 March 2021 and from 16 May 2022 to 6 Jun 2022 (the exact dates are shown by hovering with the mouse on each point of the time series) with a limited Persistence. Therefore, such outliers are to be surely considered as normal ones due to the presence of dirty particles that disappear after some days. The same applies to all other pixels reported as Outliers in the color map on the top part of the Pin, the one in contact with the wafer and subject to rubbing (not shown here for the sake of brevity). Therefore, one can conclude that no alert is given at the final date of the study concerning possible failures of the Bottom left Pin.

**Figure 10:**
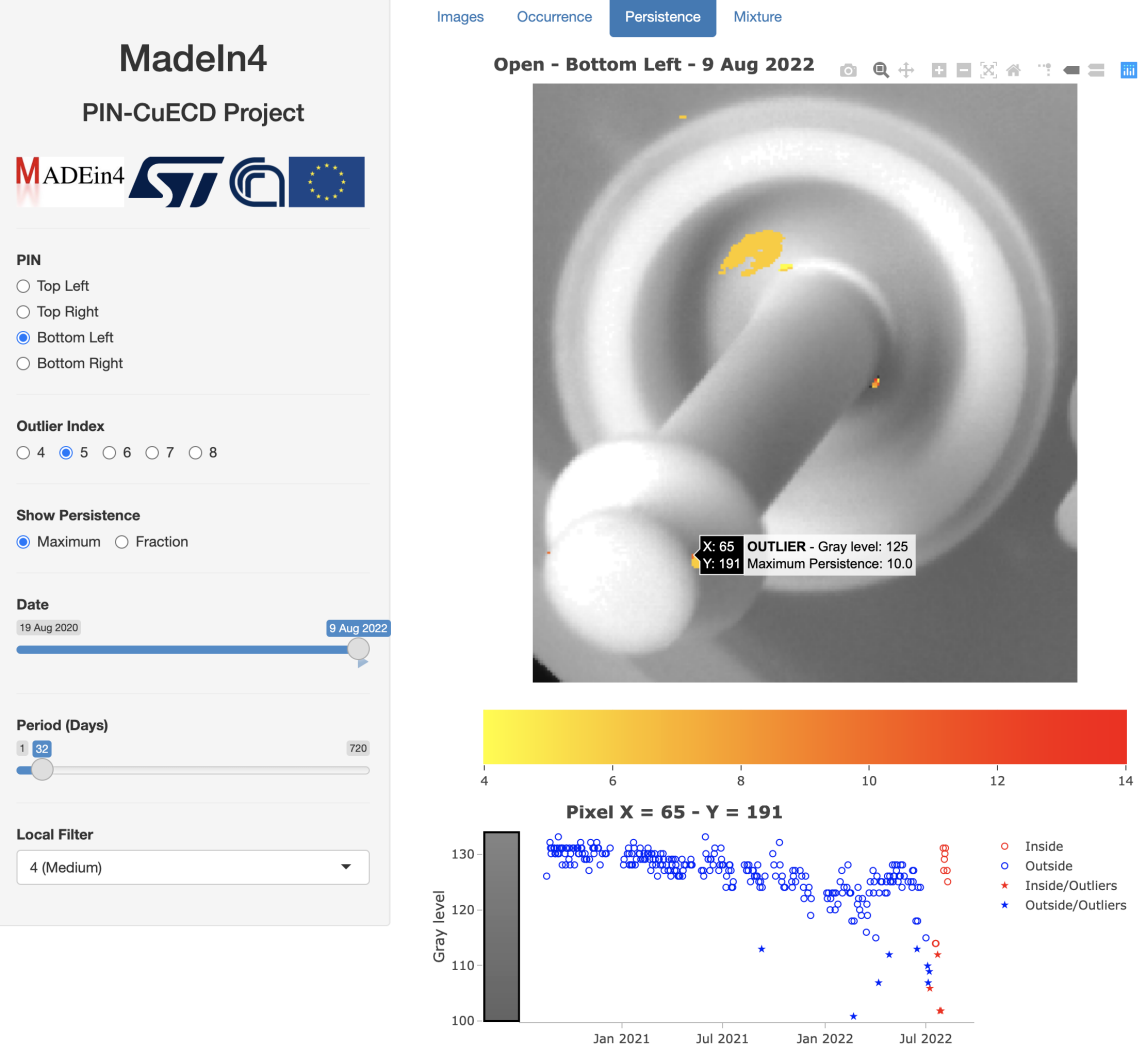
Map of Persistence through the tab Persistence. Image refers to the Bottom left Pin for a temporal range 8 July 2022 to 9 August 2022 and values of the Outlier index O≥6. Source: own research.

Figure 9 covers the entire time span of the study and was obtained for illustrative purposes of the present paper to show the location of all artifacts along the time span. In practical cases, one is interested in artifacts present only recently, discarding the ones present in previous periods. This can be easily obtained by selecting in the left menu of the graphical window a smaller Period (e.g., 30 days) and a final date as the last available one. In this way, the color map is shown only for those pixels that are outliers in the selected time period (in example of Figure 10 the window is 30 days ending with the last day of the period). The full time series, including previous outliers eventually, is therefore shown by selecting a pixel of the Pin image—the points of the time series outside the temporal range will be colored blue (see Figure 10 with the time series shown for the pixel of coordinates x=65, y=191).

Incidentally, Figure 10 reveals the presence of droplets at the base of the Pin during the last 30 days of the study. Again, Figure 10 does not indicate long Persistence prone to a rupture of the Pin at the end of the study.

**Figure 11:**
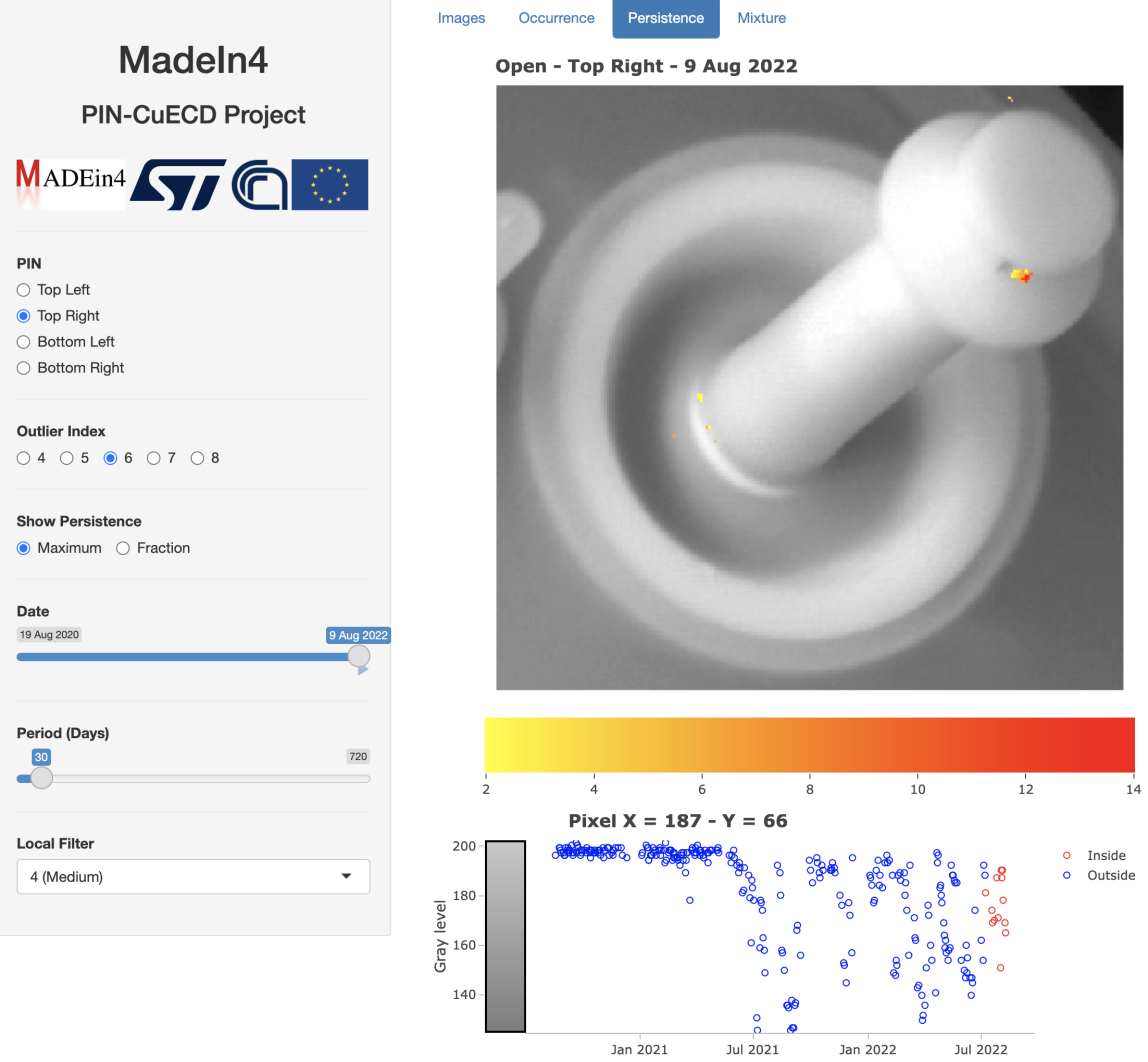
Map of Persistence through the tab Persistence. Image refers to the Top right Pin for a temporal range 8 July 2022 to 9 August 2022 and values of the Outlier index O≥6. Time series of the gray level is shown for the pixel of coordinates x=187, y=66. Source: own research.

Figure 11 refers to the Top right Pin for the last 30 days of the study and Outlier index O≥6.

It reveals the frequent presence of dirty particles (black pixels) around coordinates x=187, y=66 from the end of June 2021 up to the last days of the study (August 2022). In practice, one can observe a strong Persistence with long duration which indicates a high probability of dirty particles to adhere to the Pin and a greater resistance to be washed out by cleaning processes, conditions that are exactly prone to a progressive increased friction of that part of the Pin and ultimately to the degradation of the Pin. Direct visual inspection of the Pin from STMicroelectronics confirms the ongoing process of degradation.

Note that in the time series, no data are labeled as Outliers at the pixel of coordinates x=187, y=66. This is due to the fact that the anomaly condition became so frequent within the two years that it cannot be considered an Outlier anymore from a statistical point of view.

## 6. Discussion

A camera system endowed with an image processing software has been developed to monitor Pins that hold a wafer in the growth chamber during normal operations for the production of semiconductor devices. The system aims at using a PdM strategy to monitor the Pins to avoid unnecessary maintenance interventions and ruptures of Pins that increase the downtime of the production.

A 2-year study was conducted on a prototype system with one optical sensor and images taken on a nonregular basis.

The study allowed for reaching the following objectives:

- To understand the artifacts present in the images (e.g., dirty particles, droplets);
- To select the artifacts that are indicators of a rupture of the Pin (dirty particles, identified by dark pixels);
- To give an indication of the mechanisms that give rise to the rupture of the Pin (rubbing of the Pins against the wafer);
- To locate the Pins with an ongoing significant degradation process prone to a future rupture.

A highly interactive visualization tool has been developed to monitor the Pins and highlight artifacts. The tool is the result of a statistical methodology able to estimate artifacts on a pixel by pixel basis. A pre-processing of the images has been necessary to generate subimages of the Pins, crop them, register all of them by a full six-parameter affine matrix, and equalize using a Histogram Matching algorithm.

First of all, the analysis of images gave a clearer picture of the type of artifacts that have to be expected on the Pin. In addition, indications on the random variability of images (not due to external situations as ordinary and extraordinary maintenance) were obtained. Artifacts on the Pins can be of different types. Some of them cannot be distinguished by image processing tools alone (e.g., dirty particles and cuts on the Pins, the latter being the cause of the failure of the Pin and therefore of the entire system). Therefore, some special indicators have been developed based on the persistence of the artifacts, arising from the rationale that artifacts due to normal conditions have a limited, yet long, duration in time, whereas the ones leading to a failure are permanent.

This experience allowed one to better tailor the parameters of the methodology, called for more advanced, accurate tools, and gave indications on some problems of the camera/environment part of the system to be addressed in future releases.

Currently, the algorithms developed are capable of catching anomalies, effectively transforming the maintenance approach from preventive to predictive. Furthermore, it is possible to easily analyze the condition of Pins along the time distinguishing false alarms due to water drops or particles deposited on the Pin.

The main results of the study have been to detect the Pin (Top right) prone to a rupture, while the other ones did not show precursor signs at the end of the study. From a practical point of view, the time of replacement of the Pins has been increased by a factor at least 2, reducing downtime.

Indications for further research include:

- To ease and make more reliable the registration of the subimages of the Pins by inserting some sign of identification in key locations of the Pin, e.g., engraving in a visible way some particular sign. This is useful also when the Pins of the chamber undergo little independent movements during maintenance operations;
- To develop a methodology to classify artifacts, recognized through Outliers in the present study, to automatically discard some unwanted ones (e.g., droplets), possibly using tools from Deep Networks. This would replace use of Outliers for detecting anomalies, and as a consequence, remove the limitation of the present methodology that an artifact must be present for a shorter time than the normal status (see Figure 11);
- From the hardware point of view, to improve the camera system by better focusing the images;
- To use two, or better, four dedicated cameras;
- To take pictures regularly on a daily basis;
- To avoid as much as possible disassembling and reassembling the camera during ordinary maintenance.

## Figures and Tables

**Table 1:** Specificatons of the Teledyne FLIR FS-PGE-50S5M-C camera. Source: https://www.flir.it/products/blackfly-s-gige/?model=BFS-PGE-50S5M-C, accessed on 22 May 2023.

Feature	Specs
Resolution	2448 × 2048
Frame Rate	24 FPS
Megapixels	5.0 MP
Chroma	Mono
Sensor	Sony IMX264, CMOS, ${2/3}^{\prime \prime }$ Global shutter
Readout Method Pixel Size	3.45 μm
Lens Mount	C-mount
ADC	12-bit
Minimum Frame Rate	1 FPS
Gain Range	0 to 48 dB
Exposure Range	13 μs to 30 s
Acquisition Modes	Continuous, Single Frame, Multi Frame
Partial Image Modes	Pixel binning, decimation, ROI
Image Processing	Gamma, lookup table, and sharpness
Sequencer	Up to 8 sets using 2 features, exposure and gain
Image Buffer	240 MB
Power Consumption	3W maximum
Dimensions/Mass	29 mm × 29 mm × 30 mm / 36 g
Machine Vision Standard	GigE Vision v1.2

**Table 2:** Monthly frequency of available images. Source: own research.

Month	Images	Month	Images	Month	Images
August 2020	5	August 2021	10	August 2022	7
September 2020	20	September 2021	9		
October 2020	17	October 2021	8		
November 2020	12	November 2021	11		
December 2020	3	December 2022	4		
January 2021	14	January 2022	12		
February 2021	17	February 2022	11		
March 2021	18	March 2022	13		
April 2021	14	April 2022	14		
May 2021	8	May 2022	15		
June 2021	13	June 2022	10		
July 2021	10	July 2022	10		

## Data Availability

The data of this study are not publicly available because proprietary of STMicroelectronics and classified.

## Supplementary Materials

The following supporting information can be downloaded at https://www.mdpi.com/article/10.3390/s23146249/s1, Video S1: video-10202020152446-0000.mp4; Video S2: imPINseqO_BLr.mp4; Video S3: imPINseqO_BRr.mp4; Video S4: imPINseqO_TLr.mp4; Video S5: imPINseqO_TRr.mp4.

## Abbreviations

The following abbreviations are used in this manuscript:

AI	Artificial Intelligence
CLAHE	Contrast Limited Adaptive Histogram Equalization
EM	Expectation–Minimization
FPS	Frames Per Second
GMM	Gaussian Mixture Models
IQR	InterQuartile Range
ML	Machine Learning
PM	Preventive Maintenance
PdM	Predictive Maintenance
V-DoE	Virtual Design of Experiments
